# P16 - A child with solar urticaria

**DOI:** 10.1186/2045-7022-4-S1-P71

**Published:** 2014-02-28

**Authors:** Suleyman Tolga Yavuz, Mutluay Arslan, Burak Uğur Şimşek

**Affiliations:** 1 Department of Pediatrics, Gulhane Military School of Medicine, Ankara, Turkey

## Introduction

Solar urticaria is a rare photodermatosis characterized by pruritus, stinging, erythema, and wheal formation after a brief period of exposure to natural sunlight or an artificial light source emitting the appropriate wavelength. Herein, we present a Turkish boy with solar urticaria.

## Case report

A 9-year-old boy admitted our outpatient department suffering from urticarial lesions which develop only in the uncovered body areas, particularly in his hands and legs, within a short time after exposure to sunlight especially in the noon time. Hives were not triggered with exercise, sweating, hot water exposure or stress. His symptoms persisted for three months and urticarial lesions resolved spontaneously in less than one hour after cessation of sun exposure. Skin lesions were not accompanied by any other systemic symptoms. His family or personal history of atopy were not significant. His systemic physical examination and routine laboratory tests including basic auto-immune work up revealed no abnormal findings except grass pollen sensitivity in skin prick tests. He was diagnosed as solar urticaria and prescribed antihistamine and sun protective factors before exposure to sunlight particularly in the sunny days. He remained asymptomatic after 3-months follow-up with the help of preventive measures.

## Conclusion

Solar urticaria is a rare type of photodermatoses. Polymorphic light eruptions should be considered in the differential diagnosis. Taking measures to avoid or minimize sun exposure is the most important step in the management of the disorder. Premedication with antihistamines are also reported to block wheal formation and minimize pruritus.

